# Lifetime and Molecular Coupling in Surface Phonon Polariton Resonators

**DOI:** 10.1021/acsomega.4c01009

**Published:** 2024-05-02

**Authors:** S. Maryam Vaghefi Esfidani, Marko J. Tadjer, Thomas G. Folland

**Affiliations:** † Department of Physics and Astronomy, The University of Iowa, Iowa City, Iowa 52242, United States; ‡ U.S. Naval Research Laboratory,4555 Overlook Ave SW, Washington, District of Columbia20375,United States

## Abstract

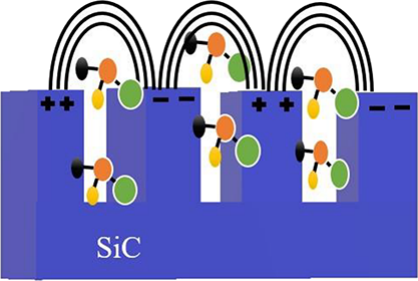

Surface phonon polariton (SPhP) modes in polar semiconductors offer a low-loss platform for infrared nanophotonics and sensing. However, the efficient design of polariton-enhanced sensors requires a quantitative understanding of how to engineer the frequency and lifetime of SPhPs in nanophotonic structures. Here, we study organ-pipe resonances in 4H-SiC trenches as a prototype system for infrared sensing. We use a transmission line framework that accounts for the field distribution within the trench, accurately predicting mode frequency and lifetime when compared against finite element method (FEM) electromagnetic calculations. Accounting for the electric field profile across the gap is critical in our model to accurately predict mode frequencies, quality factor (Q factor), and reflectance, outperforming previous circuit models developed in the literature. Beyond structural simulation, our model can provide insights into the frequency ranges in the Reststrahlen band where enhanced sensor activity should be present. The radiative lifetime is significantly enlarged close to the longitudinal optic phonon, restricting sensor efficiency at this wavelength range. This pushes the optimal frequency for sensing closer to the center of the Reststrahlen band than might be naively expected. This model ultimately demonstrates the primary challenge of designing SPhP-based sensors: only a relatively narrow region of the Reststrahlen band offers efficient sensing, guiding future designs for infrared spectroscopy.

## 1 Introduction

Surface polariton resonances with subdiffraction confinement form through the strong coupling between coherently oscillating charge and light.^1,2^ Their advantages for applications in sensing^3,4^ optoelectronics,^5−7^ and active optics^8^ arise from their ability to localize electric fields to deep subdiffraction length scales. An active area of interest is surface-enhanced infrared absorption spectroscopy (SEIRA), which allows absorption spectroscopy to detect orders of magnitude smaller concentrations of materials^9,10^ when compared with conventional infrared techniques. Surface plasmon polaritons (SPPs), which arise due to coupling between light and electrons, have been extensively studied for this application.^11,12^ Still, their relatively short lifetime (hundreds of femtoseconds) limits achievable confinement and coupling.^12−14^ This has motivated the search for longer lifetime polariton modes, including dielectric antennas,^4,15^ Tamm hybrid structures,^16^ and surface phonon polaritons (SPhPs).^8,14^ Surface phonon polaritons are particularly interesting due to their occurrence in the molecular fingerprint window from 6 to 20 μm.^17,18^ Polar dielectric materials support them and arise due to coupling to polar optical phonons and light. Of the SPhP materials, SiC is a prototypical example.^19,20^ It supports SPhP modes in the spectral range between transverse optic (TO, 797 cm^–1^) and longitudinal optic (LO, 971 cm^–1^) phonon frequencies, known as a Reststrahlen band. The lifetimes associated with SPhP modes are typically on time scales in the few to tens of picosecond range, several orders of magnitude longer than free carriers in metal.^21,22^ This results in lower loss nanophotonics, which can be directly applied to realize narrowband and efficient optoelectronics^23,24^ and sensor technologies.^25^

Given the success of prior work exploiting SPPs for SEIRA, SPhPs have also been proposed as promising for sensing.^18,26,27^ However, despite several reports of surface-enhanced sensors, performance has been comparatively limited^26,27^ compared to metal-based structures. There is no clear reason why SPhP-based sensors are less effective based on available understanding of phonon polaritons and available data. The gaps in our understanding likely arise because SEIRA^3,4,28−33^ is a resonant process; this relies on optimizing resonances so that when loaded with a molecule, the cavity becomes optimally coupled.^4^ As the mode lifetime in sensor structures made for SEIRA has largely been unexplored for SPhP modes, this is suggestive that enhancement must operate differently between plasmon and phonon-based sensors. Specifically, the highly dispersive nature and low group velocity of SPhP modes must be accounted for. Getting this insight requires a new approach to analyzing SPhP structures where mode lifetime can be calculated. It is worth noting that scattering models have been applied to individual metal antennas^34^ in the mid-infrared. However, it is more robust to consider results from collective arrays because the long wavelength of light, large arrays is typically used.^3,4,30^

**Figure 1:**
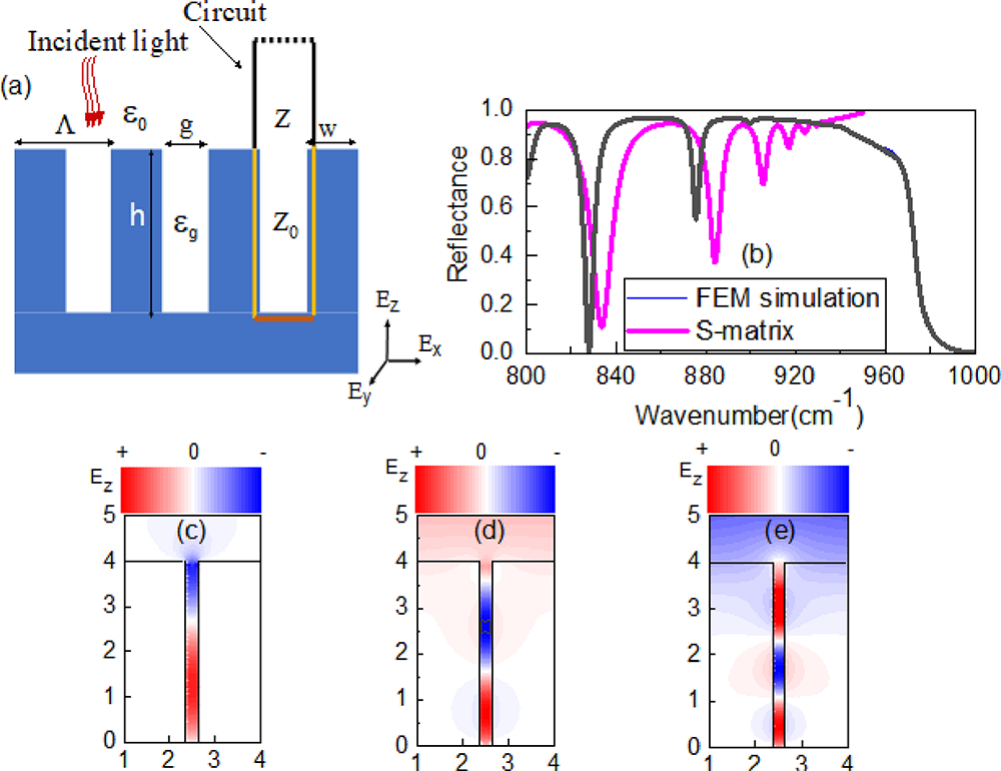
(a) Schematic for a MIM waveguide with a gap (*g*), pitch (Λ), and height(*h*). Equivalent circuits were depicted with two elements: a short transmission line section (yellow line) and a fully reflective base (red line). (b) FEM simulation reflectance spectra compared against the S-matrix model for SiC grating with *h* = 4 μm, *g* = 0.2 μm. (c–e) Corresponding simulated field profiles using FEM simulation for ω ∼ 830 cm^–1^, ω ∼ 880 cm^–1^, and ω ∼900 cm^–1^, respectively.

To perform our analysis, we choose a high aspect ratio SPhP grating as a relatively simple structure to understand the sensor performance (Figure 1a). It is defined by the physical gap (*g*), period of the structure (Λ), and height (*h*), and its simplicity is advantageous for understanding the physical mechanisms in this system. Finite element method (FEM) numerical simulations in CST Studio Suite are used to model the grating period using a frequency-domain solver with a tetrahedral mesh. To define the periodicity of the grating, a Floquet unit cell boundary condition is used on the sidewalls, and a perfectly matched layer at the bottom represents the semi-infinite nature of the SiC. FEM results reveal that this structure has a series of robust and high *Q* factor resonances in the Reststrahlen band of the structure (Figure 1b). Each resonance corresponds to the number of field maxima within the trench, which can be directly compared to the standing waves that form in a pipe. (Figure 1c–e). This type of structure has been studied for metallic trenches^35^ and SPhP modes.^18,36^ Notably, they have already been demonstrated as a platform to realize molecular strong coupling for SPhP modes.^18,37^ Prior works have broadly taken two approaches to modeling light in these structures: equivalent circuit and transmission line frameworks. Both provide a greater physical insight than the various forms of full-wave simulations, as they describe the specific nature of the photonic modes in the structure, as opposed to directly solving Maxwell’s equations. The equivalent circuit framework uses LC resonances to model electromagnetic resonances in different geometries. They were the first to investigate these structures and successfully matched mode frequencies using just one or two fitting parameters.^36^ However, they do not provide detailed information on the *Q* factor for SPhP structures, which precludes their use for analysis of SEIRA enhancement. Instead, the transmission line frameworks treat the structure as a metal–insulator–metal (MIM) waveguide array. These are more generic as they can be used to extract complete reflection spectra and typically do not require fitting parameters^35^; however, have not been used in the exploration of SERIA, including an analysis of lifetimes. The transmission line model also offers a more physically intuitive explanation for the resonances in these gratings as standing waves, which form in each waveguide, analogous to the formation of standing waves in organ pipes.

In this paper, we apply the transmission line framework and develop both a cavity model and an S-matrix model for SPhP supporting trenches, providing a physical insight into modes and SEIRA in the structure. Crucially, we show that the radiative behavior of SPhP modes restricts the efficacy of SPhP sensors. Our model extends prior work by explicitly including the field distribution within the trench, which is critical for understanding the mode lifetime of these structures. Our model can match mode frequencies within 1% and *Q* factors within 50% or better for all modes inside the Reststrahlen band when compared to FEM simulations. Our lifetime analysis can also broadly predict the most sensitive regions of the Reststrahlen band for sensor operation. Counterintuitively, we show that only a fraction of the Reststrahlen band will show substantial enhancement factors, and counterintuitively, this region is not close to the LO phonon where confinement is most significant. The simplified models of the MIM waveguide demonstrated in this work hold great promise for predicting the SPhP modes and *Q* factor for characterizing nanophotonic media in the mid-infrared region. Crucially, this indicates a narrower design space for SPhP-based sensors, which our tools can help address.^36,38^

## 2 Methods

In our approach, we leverage a transmission line framework that extends work on gold trench arrays.^35^ Following the approach of prior work, we first can define the real and imaginary parts of propagation constant (β) inside each trench in the structure following the MIM waveguide dispersion relation^18,38,39^:
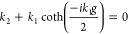
1Here, the in-plane wave vector, *k*_*i*_, is defined by
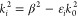
2and ε_*i*_ is the dielectric function of each layer. For the dielectric function of silicon carbide, we use the harmonic “TOLO” formalism:
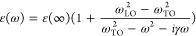
3where ω_TO_ = 797 cm^–1^ and ω_LO_ = 971 cm^–1^ correspond to the TO and LO phonon frequencies and γ = 4 cm^–1^ and ω are the phonon’s damping constant and frequency of the excitation, respectively.^40,41^

We then use this propagation constant found from the MIM dispersion to define the impedance of the wave within the trench. From Kirchhoff’s law, in the quasi-static limits, the impedance inside each slit can be accounted for by the ratio between voltage applied (*v*) and current (*I*) at two ports^35,42^
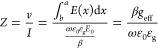
4Here, ε_g_ is the dielectric permittivity of the material in the gap. In an earlier work,^35^ the electric field distribution, *E*(*x*), is assumed to be constant within the trench and zero elsewhere, making *g*_eff_ = *g*. However, in our models, the electric field distribution within the waveguide has been explicitly integrated as part of the impedance calculation. This integral over the electric field provides an “effective gap (*g*_eff_)” typically larger than the physical gap (*g*), assuming a constant electric field, dramatically changing the impedance closer to the LO phonon. Throughout the manuscript, we will compare our results for a constant field (*g*_eff_ = *g*) and a varying field (*g*_eff_ ≠ *g*) to show that treating the variable field is required for SPhP-based structures.

To simulate the coupling of free space light to each trench, we need to calculate the impedance of the wave in free space when incident on a grating. This allows us to calculate the Fresnel coefficients for coupling between waves in the trench and free space. For a transverse magnetic (TM) wave incident at an angle θ, the characteristic impedance of the input medium per unit height (*Z*_0_) at an angle θ is defined by^35^
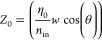
5Here, *w* is the period,  is the refractive index, and  is vacuum impedance, where μ_0_ and ε_0_ are the dielectric permeability and permittivity in vacuum, respectively, and ε_in_ is the permittivity of the input medium. All simulations in our paper assume a normal incident angle of light (θ = 0).

We can then use the above impedances to calculate the system’s properties. Specifically, we use two approaches: S-matrix and cavity models. The scattering matrix (S-matrix) model efficiently simulates the reflectance spectra for SPhP modes in the trenches. It is based on a set of normalized S-matrices, accounting for the propagation constant and impedance of the waves inside the structure and in free space.^43,44^ The trench is considered a short transmission line and a fully reflective () base, as shown in Figure 1a. The overall S-matrix and reflectance of this short section is given by^43,45^
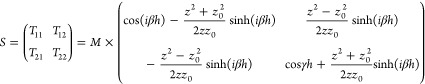
6
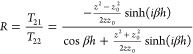
7

**Figure 2:**
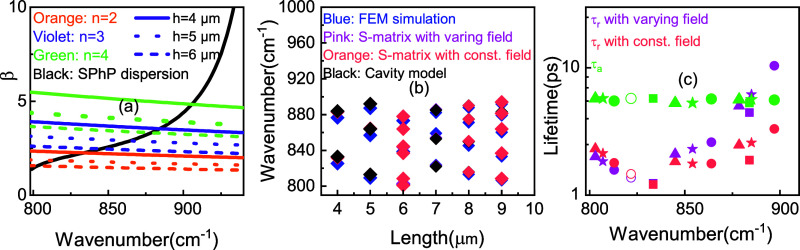
SiC grating with *g* = 0.2 μm, Λ = 5 μm. (a) Calculated mode positions of standing waves in SiC grating with various heights using the cavity model. (b) Calculated SPhPs for the grating with heights ranging from 4 μm to 10 μm using two simplified models and FEM simulation. (c) Calculated radiative lifetime (τ_r_) with varying and constant field vs absorption lifetime (τ_a_) for SiC grating with heights ranging from 4 μm to 6 μm. (square: *h* = 4 μm, hollow circle: *h* = 4.5 μm, circle: *h* = 5 μm, star: *h* = 5.5 μm, triangle: *h* = 6 μm).

Our cavity model takes a more simplified approach. The cavity model considers the trench an open-ended pipe in which the modes propagate with propagation constant β*.* The frequency of the modes is defined by the condition for standing waves in an open-ended pipe, expressed in terms of the propagation constant (*Re*(β)) as^18^
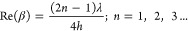
8where *n* is the number of antinodes of a standing wave in an open-ended pipe and is defined as an integer and λ is the free-space wavelength. We note that for a weakly decaying wave, the wavelength is determined by the real part of β, representing the wavelength of the mode. The imaginary part, which is typically smaller, corresponds to absorption processes within the waveguide. Therefore, when we evaluate the frequency of SPhP modes in the cavity, we only account for the real part of β, as in eq 8. SPhP modes are subsequently found by evaluating the intersection of the standing wave condition (eq 8) and the numerical dispersion relation for β found by solving eq 2, shown in Figure 2a. The imaginary part is then exclusively used in the calculation the absorption lifetime (τ_a_) for each mode with frequency defined by the real part of β and eq 8. The lifetime of these modes is then calculated using the radiative lifetime (τ_r_) and absorptive (τ_a_) of a standing wave in a cavity, using the following definitions^18^:
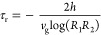
9
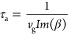
10

To account for waveguide losses in the absorption lifetime, the imaginary part of β in eq 2 is considered. Here, *v*_g_ is the group velocity, and *R*_1_ and *R*_2_ are the power reflection coefficients from the cavities’ top and bottom that can be defined by the Fresnel equations^18,35^:
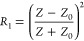
11
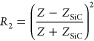
12where *Z*_SiC_ can be calculated by substituting *n*_in_ with the refractive index of SiC (*n*_SiC_) in eq 5. Unlike in prior works, the reflectance values are defined by the impedance of the waves in the structure, which is critical for accurately calculating reflection coefficients in this model. The total mode lifetime (τ) and reflectance for each resonance for the cavity model can then be calculated from the temporal coupled wave theory^18,46−48^
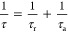
13
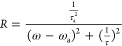
14

The *Q* factor of cavity resonances associated with lifetime can be written as^49^
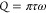
15

## 3 Results

We choose parameters for the grating close to those used in past works to assess the model’s validity, selecting *g* = 0.2 μm and Λ = 5 μm, while varying the heights of the grating.^18^ Multiple modes and heights are plotted against each other to encompass the general trends as the modes tune with cavity length. The physical interpretation for this can be seen in Figure 2a, with a small change in the cavity length, the modes also tune over a narrow range. In this way, we can systematically plot mode properties over a wide range of frequencies using the cavity model. Furthermore, we compare our transmission line models (S-matrix and cavity) to FEM simulations. First, we track the frequency of the resonant modes in the structure as a function of height (Figure 2b). Our two approaches agree with FEM simulations within 1% for all modes that we consider in the Reststrahlen band. The results show the significant dependence of SPhP modes on the grating height (*h*). More resonances appear as the grating becomes deeper over the spectral range of SiC in the Reststrahlen band, and more standing waves form in the trench while increasing the grating height. Our results agree with past work and highlight the accuracy of the model.^18^ We note that, unlike the previous works, no fitting parameters have been used to achieve this fit; all values are purely based on the dielectric function. We also calculate the lifetime for various heights using our cavity model, as shown in Figure 2c, based on eqs 9 and 10. Our results show that while the absorptive lifetime (τ_a_) remains constant within the Reststrahlen band, the radiative lifetime (τ_r_) varies drastically. Consequently, the radiative lifetime becomes the deciding factor in the quality factor and reflectance of the resonators at different frequencies across the Reststrahlen band and is a minimum at 830 cm^–1^ for this range of gratings. From eq 14, we anticipate that this also represents the minimum of observed reflection.

**Figure 3:**
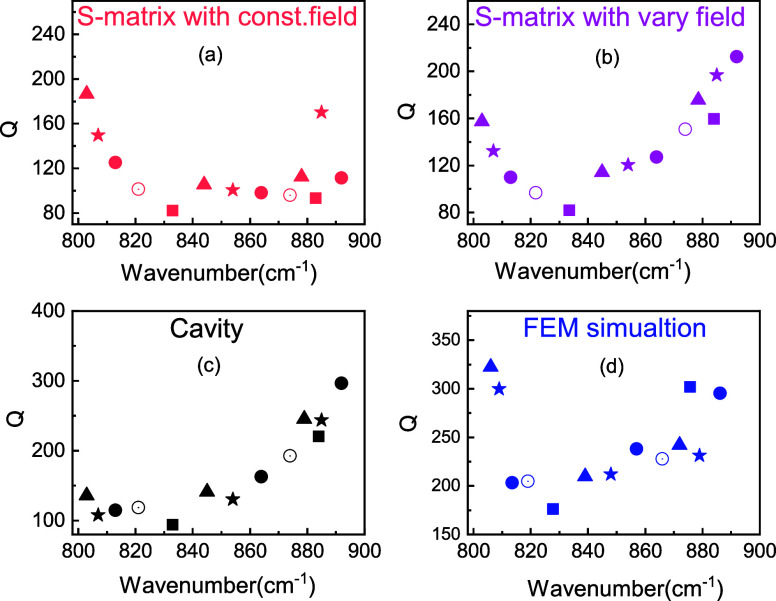
Calculated *Q* factor for SiC grating with *g* = 0.2 μm, Λ = 5 μm, and various heights (square: *h* = 4 μm, hollow circle: *h* = 4.5 μm, circle: *h* = 5 μm, star: *h* = 5.5 μm, triangle: *h* = 6 μm): (a) S-matrix with constant field. (b) S-matrix with varying field. (c) Cavity model. (d) FEM simulation. All three models (FEM simulation, cavity model, and S-matrix with varying fields) are in agreement and follow the same trends. This suggests that simplified models are promising for efficient control over optical response.

A significant advantage of our transmission line models is that we can accurately predict the modal *Q* factor. The *Q* factor, given by ,^50^ represents the mode line width and is directly related to the balance between radiative and absorptive losses of a given mode. In the cavity model, the value of the *Q* factor can be calculated through eq 15, and it can be obtained from a Lorentzian fitted to reflectance spectra for S-matrix and FEM simulation. The *Q* factor for different modes and multiple heights is shown in Figure 3, where we use the different heights to tune the modes across a range of frequencies within the Reststrahlen band. The modes observed in SiC exhibit a narrow bandwidth and calculated *Q* factor by FEM simulation ranging from 176 to 322 like those previously reported.^51,52^ The corrected impedance allows us to calculate the *Q* factor for all modes in the Reststrahlen band within 50% or better, and there is a noticeable difference close to LO in values and trends for varying and constant field compared with FEM simulation. Crucially, without the correction for the varying field, we could not predict a general trend of the *Q* factor decreasing to a minimum and then increasing with frequency. That is because, for a polarization-dependent MIM waveguide, a significant amount of the energy resides inside SiC.^52^ Deviations between the FEM simulation and our transmission line models are likely due to lifetime effects arising from scattering at the entrance to each groove, which is not considered in our simulations. We note some scatter on the data due to variations in the radiative lifetime with heights. According to eq 9, the greater height would increase the radiative lifetime, leading to changes in reflectivity and a larger *Q* factor. However, this slight variation in radiative lifetime allows us to see general trends, indicating that the region with the lowest *Q* factor is close to 830 cm^–1^.

**Figure 4:**
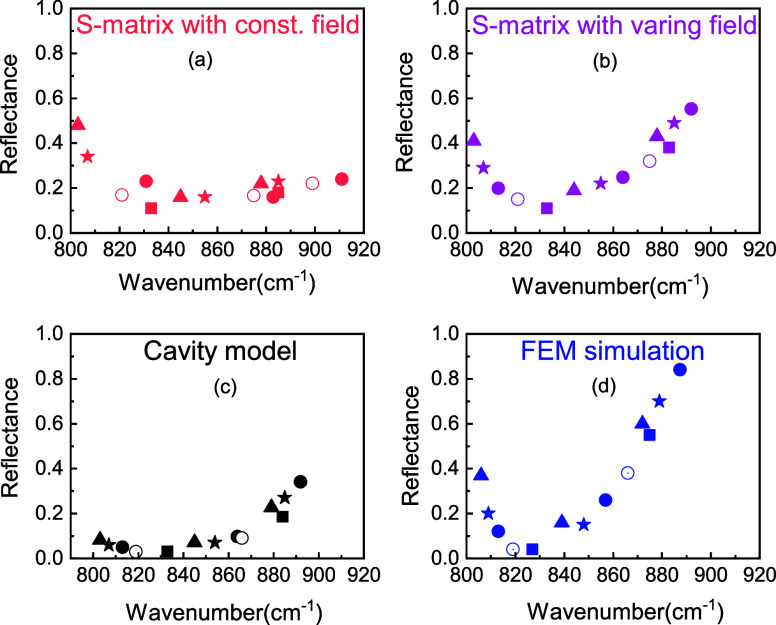
Calculated reflectance for SPhP modes formed in SiC grating with *g* = 0.2 μm, Λ = 5 μm, and various heights (square: *h* = 4 μm, hollow circle: *h* = 4.5 μm, circle: *h* = 5 μm, star: *h* = 5.5 μm, triangle: *h* = 6 μm): (a) S-matrix with constant field. (b) S-matrix with varying field. (c) Cavity model. (d) FEM simulation.

We are also able to calculate the reflectance minima given by eqs 7 and 14for the S-matrix and cavity models, respectively, to better analyze the grating interaction with light (Figure 4). To use eq 14, we only consider the on-resonance reflectance, aka ω = ω_a_. The effect of corrected impedance allows us to calculate reflectance within an absolute error of 0.29 or better for all modes considered here and again track the general trend of a minimum in the reflectance at approximately 830 cm^–1^. Unlike the *Q* factor, which describes the decay pathways in the mode, reflectance indicates the interaction between radiative and absorptive losses. From eq 14, we note that the minimum in reflectance shows the point where the cavity lifetime is a minimum, which agrees well with simulations for the *Q* factor (Figure 4) and lifetime (Figure 2c). Away from this point, the balance of lifetimes begins to favor reradiation, not absorption of light.

**Figure 5:**
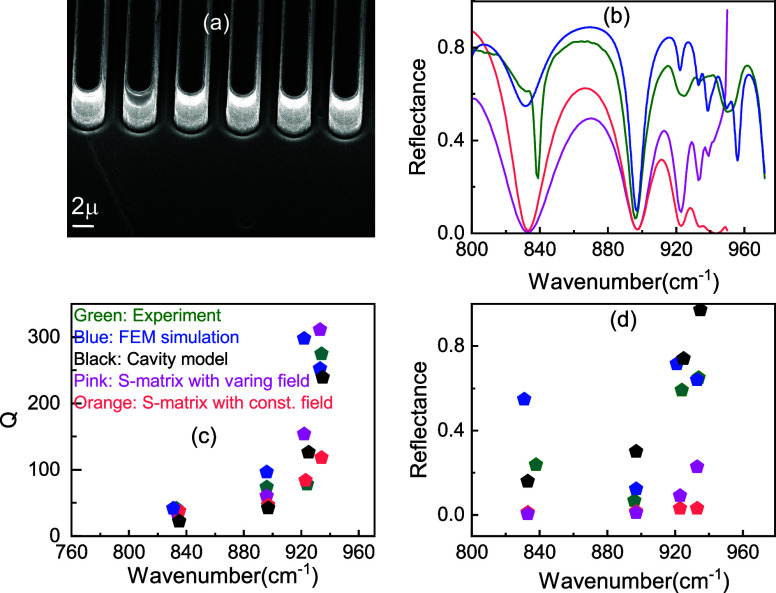
(a) SEM image for a grating height and pitch of 11.5 μm and 5 μm, respectively. (b) Experimentally measured reflectance spectra are compared against FEM and transmission line simulations. (c, d) Calculated *Q* factor and reflectance for SPhP modes. Strong and weak SPhP modes are predictable using the FEM simulation and transmission line framework, and they follow the same trend as the resonant modes obtained from FTIR microscopy.

To validate our model, we experimentally measure the reflection spectra of deep grating with a constant fill fraction (*g*/Λ ) of 0.5 to theoretically predict the spectral behavior. Here, a 4H-SiC grating with Λ = 5 μm and *h* = 11.5 μm (see ref (19)) is fabricated. To do so, contact photolithography was used to define the grating structures in photoresist. An etch mask was then deposited consisting of e-beam deposited Cr(10 nm)/Au(100 nm) with electroplated Ni(1600 nm) to ensure stability during etching. After liftoff, deep reactive-ion etching (DRIE) is used to etch SiC for 2 h with SF_6_/O_2_ inductively coupled plasma, using a κ-etch process described in references.^53,54^ A scanning electron microscope (SEM) image of the representative arrays with a tilted angle of 30° shows that dry etching causes a gap between trenches to change with height, resulting in a taper (Figure 5a). We measured the reflectance spectrum using FTIR microspectroscopy (Bruker Vertex 70v) using a near-normal objective, with the light oriented perpendicular to each trench, exciting the TM mode. To model the reflection behavior of such a structure, we perform FEM electromagnetic simulations. Our FEM simulation allows us to use the exact geometric parameters for the trench. It includes the effect of taper across the height to make the geometry match the fabricated structure as closely as possible. Our FEM simulation can predict all SPhP modes seen in the experiment. The SPhP modes are also predictable using the transmission line model by taking the gap size as the average between the top and bottom of the grating (Figure 5b). As demonstrated in our prior simulations, correcting impedance in our transmission line model allows us to better match the strong and weak resonances in the Reststrahlen band. Note that by considering the electric field across the structure, spectra are qualitatively close to experimental data for high-order localized modes. We have further analyzed the *Q* factor and reflectance for the resonant modes, and our results are in qualitative agreement with the experimental data and follow the same trend (Figure 5c, d). Following our prior results, the corrected impedance data matches the experimental data better. However, due to limitations of the impedance model, which assumes that the gap is small compared to the grating period, the *Q* factor and reflectance differ. This can also be attributed to the nonvertical sidewalls in the structure, which will naturally alter the impedance. We also note that the mismatch for the lowest frequency mode, close to TO phonon of SiC, which arises from the fact that we do not consider the coupling of SPhP modes to zone-folded LO phonons. These are a result of the stacking order in the atomic lattice in the *c*-axis and the SPhPs in SiC.^55,56^

## 4 Discussion

**Figure 6:**
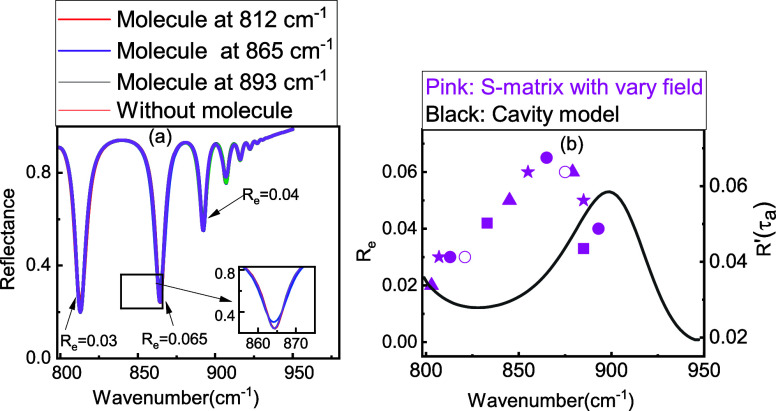
(a) Coupling between SPhP modes and molecule vibration for SiC grating with *h* = 5 μm and *g* = 0.2 μm using the S-matrix model. (b) SEIRA enhancement calculated for the test molecule, SiC grating with *g* = 0.2 μm and various heights using the S-matrix model (square: *h* = 4 μm, hollow circle: *h* = 4.5 μm, circle: *h* = 5 μm, star: *h* = 5.5 μm, triangle: *h* = 6 μm) and cavity model (black line)

One of the significant advantages of our models is they allow us to better understand the behavior of SPhP modes in sensor structures. The grating behaves as an effective SEIRA sensor if we design it to maximize the changes in reflectance upon introducing a molecule. To provide the necessary understanding of SEIRA sensors made from gratings, we simulate a dummy molecular vibrational band located at the frequency for each SPhP mode for a grating with *h* = 5 μm and *g* = 0.2 μm, using the S-matrix model (Figure 6a). The parameters for the molecular vibrations, such as the damping constant and absorption coefficient, are γ = 8 cm^–1^ and α = 50 cm^–1^, respectively, based on those used in a prior work for cyclohexane.^18^ Therefore, at resonance, subtle changes become observable in the reflectance spectra, and by analyzing these changes, we can assess the SEIRA sensitivity of the structure. To evaluate the sensing performance of each mode in the structure, we consider the enhancement on resonance (*R*_*e*_ = *R*_molecule–SPhPs_ – *R*_SPhPs_). In each case, we tune the molecular resonance to the center of the SPhP mode to capture information about each mode across the full Reststrahlen band and consider multiple heights varying from 4 to 6 μm, as shown in Figure 6b. Remarkably, we find that the maximum enhancement is observed around 860 cm^–1^, neither close to the LO phonon (where field confinement is highest) or near 830 cm^–1^, where reflectance is lowest.

To better understand this result, we can use our cavity model and results from the coupled wave theory. A SEIRA sensor works such that when we add the molecule, τ_a_ will decrease due to increased molecular absorption, thereby changing the reflectance. To find the condition that maximizes this change, we can calculate the enhancement that is derivative of reflectance (eq 14) with respect to τ_a_, which can be written as
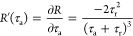
16

As shown in Figure 2c, τ_a_ is almost constant across the Reststrahlen band, and so τ_r_ is the deciding factor in the performance of the sensor at different frequencies. To find the condition within the Reststrahlen band that gives the best sensor performance, we can use the condition , which gives that when the radiative lifetime is twice the absorption lifetime (τ_r_ = 2τ_a_), which gives the maximum enhancement close to 900 cm^–1^. Therefore, this is the optimal condition to maximize the sensor’s performance.

We compare the change in reflectance from the S-matrix model (*R*_*e*_) to *R*′ (τ_a_) from the cavity model in Figure 6b. Overall, both the cavity and S-matrix models predict an increasing sensitivity in the center of the Reststrahlen band, suggesting that this interpretation of the SEIRA performance is at least qualitatively correct. However, there is a slight discrepancy in the frequency of the maximum enhancement. We attribute this to the cavity model being an oversimplification of the problem, which systematically underestimates both the *Q* factor and reflectance, as seen in Figure 3 and Figure 4. Given that the propagation losses are the same between the two model results, we anticipate that this is due to a systematic underestimation of τ_r_, likely due to phase delays associated with the reflection at the interfaces. Such delays will be included in the more robust S-matrix models. These results show the importance of τ_r_ in understanding the design of SEIRA sensors. The validity of eq 16 also suggests a couple of interesting conclusions. The equation suggests that structures with smaller τ_a_, aka larger losses, can increase *R*^′^(τ_a_) and the performance of SEIRA sensors. This counterintuitively suggests that many low *Q* factor nanophotonic structures should outperform high *Q* structures, which could explain discrepancies in the performance of plasmonic-based (low *Q* but high sensitivity) and SPhP-based (high *Q* but low sensitivity) sensors.

## 5 Conclusions

This work focuses on understanding the design of high aspect ratio 4H-SiC organ trenches supporting SPhP modes. To do so, we develop S-matrix and transmission line models using an effective gap as the new approximation to calculate the impedance of waves inside the grating. For both simplified models, the *Q* factor and absorbance are in good agreement with FEM simulations. We can use our model to accurately predict the SPhP resonances and verify that our model can reproduce the key features from experimentally measured structures. One of the main advantages is that the absorption and radiative lifetime associated with resonances can be predicted using the cavity model. We can use this to indicate the frequency range for optimal sensing in the Reststrahlen band. Our model highlights that SPhP resonances offer a relatively limited design window for the realization of SEIRA, which underpins the challenges in realizing SEIRA sensors. In particular, the long lifetimes associated with SPhPs can result in suboptimal sensor operation. Our results will accelerate the design of SPhP-based sensors, which are of growing interest to the photonics community and could be applied to other nanophotonic materials.

## Data Availability

Data and codes are available through Iowa Research Online repository, DOI: dx.doi.org/10.25820/data.006951.
